# Neutrophil-to-Lymphocyte Ratio Is an Independent Risk Factor for Coronary Artery Disease in Central Obesity

**DOI:** 10.3390/ijms24087397

**Published:** 2023-04-17

**Authors:** Zsolt Bagyura, Loretta Kiss, Árpád Lux, Csaba Csobay-Novák, Ádám L. Jermendy, Lívia Polgár, Ádám G. Tabák, Pál Soós, Zsolt Szelid, Béla Merkely, László Kőhidai, Éva Pállinger

**Affiliations:** 1 Heart and Vascular Center, Semmelweis University, Városmajor utca 68, H-1122 Budapest, Hungary; 2 Department of Public Health, Faculty of Medicine, Semmelweis University, Nagyvárad tér 4, H-1089 Budapest, Hungary; 3 Department of Internal Medicine and Oncology, Faculty of Medicine, Semmelweis University, Korányi S. u. 2/a, H-1083 Budapest, Hungary; 4 UCL Brain Sciences, University College London, 1-19 Torrington Place, London WC1E 6BT, UK; 5 Department of Genetics, Cell- and Immunobiology, Semmelweis University, Nagyvárad tér 4, H-1089 Budapest, Hungary

**Keywords:** neutrophil granulocytes, inflammation, coronary artery calcification, atherosclerosis, neutrophil-to-lymphocyte ratio

## Abstract

Several inflammatory biomarkers were found to be associated with an increased risk of cardiovascular disease. Neutrophil-to-lymphocyte ratio (NLR) is a marker of subclinical inflammation that increases with the stress response. Visceral adiposity index (VAI) calculated as a combination of anthropometric and metabolic parameters reflects both the extent and function of visceral adipose tissue. Given the association of subclinical inflammation with both obesity and cardiovascular diseases, it is plausible that the inflammation–CVD association is modulated by the amount and function of adipose tissue. Thus, our aim was to examine the association between NLR and coronary artery calcium score (CACS), an intermediate marker of coronary artery disease in asymptomatic patients across VAI tertiles. Methods: Data from 280 asymptomatic participants of a cardiovascular screening program were analysed. In addition to the collection of lifestyle and medical history, a non-contrast cardiac CT scan and laboratory tests were performed on all participants. Multivariate logistic regression was conducted with CACS > 100 as the outcome and with conventional cardiovascular risk factors and NLR, VAI, and NLR by VAI tertile as predictors. Results: We found an interaction between VAI tertiles and NLR; NLR values were similar in the lower VAI tertiles, while they were higher in the CACS > 100 in the 3rd VAI tertile (CACS ≤ 100: 1.94 ± 0.58 vs. CACS > 100: 2.48 ± 1.1, *p* = 0.008). According to multivariable logistic regression, the interaction between NLR and VAI tertiles remained: NLR was associated with CACS > 100 in the 3rd VAI tertile (OR = 1.67, 95% CI 1.06–2.62, *p* = 0.03) but not in the lower tertiles even after adjustment for age, sex, smoking, history of hypertension, hyperlipidaemia, and diabetes mellitus, as well as high-sensitivity C-reactive protein. Our findings draw attention to the independent association between subclinical, chronic, systemic inflammation and subclinical coronary disease in obesity.

## 1. Introduction

Cardiovascular disease (CVD) is one of the main causes of death, accounting for over 4 million deaths per year in the European Union [1]. Atherosclerosis is a multifactorial, systemic process leading to clinical manifestations of CVD. Atherosclerotic plaques in the coronary arteries and their calcification are hallmarks of atherogenesis and the basis of the development of coronary artery disease (CAD). Low-dose non-contrast cardiac computed tomography (CT) is a commonly used method for quantifying the extent of coronary calcification. It provides the coronary artery calcium score (CACS), a reliable surrogate of atherosclerosis. CACS above 100 is an accepted marker of moderate to high CAD risk [2,3].

Visceral adiposity index (VAI) is a marker of dysfunctional visceral adipose tissue that combines both anthropometric and functional parameters. It is calculated from body mass index and waist circumference, as well as triglyceride (TG) and high-density lipoprotein cholesterol (HDL-C) levels [4]. It shows an association with cardiovascular events [5,6,7]. Previously we reported that VAI tertiles were associated with calcium score, and the highest VAI tertile was an independent predictor for the presence of CACS > 100 in males [8].

Chronic inflammation may be characterized by the accumulation of leukocytes in the affected tissue. Although neutrophils are considered key players in acute inflammation, more and more data show that they also have a role in the maintenance of chronic inflammation. Continuously recruited neutrophils release inflammatory mediators (such as serine proteases and neutrophil extracellular traps) and also participate in the cooperation between immune cells. Quantitative and functional changes of immune cells have prognostic value in chronic inflammation. For instance, an increased number of neutrophils [9] and also a decreased number of lymphocytes are related to mortality in heart failure [10] and other cardiovascular diseases [11].

The neutrophil-to-lymphocyte ratio (NLR), which is the number of neutrophils divided by the number of lymphocytes, is a sensitive marker of inflammation that increases in response to stress. Similarly to other markers of subclinical inflammation, several studies indicate a relationship between NLR and central obesity [12,13]. Furthermore, systematic reviews, meta-analyses, and various recent studies support that neutrophil-to-lymphocyte ratio is associated with incident cardiovascular disorders, including CAD, acute coronary syndrome, and stroke, and that NLR has prognostic impacts on mortality, recurrent cardiovascular events, and even acute limb ischemia [14,15,16,17,18,19,20].

Different cut-off values may be applied according to the clinical condition and population, as has been observed in various studies [21,22].

Given the association of subclinical inflammation with both obesity and cardiovascular disease, it is plausible that the inflammation–CVD association is modulated by the amount and the function of adipose tissue. Thus, we aimed to examine the association between NLR and CACS in asymptomatic patients across VAI tertiles.

## 2. Results

**Figure 1:**
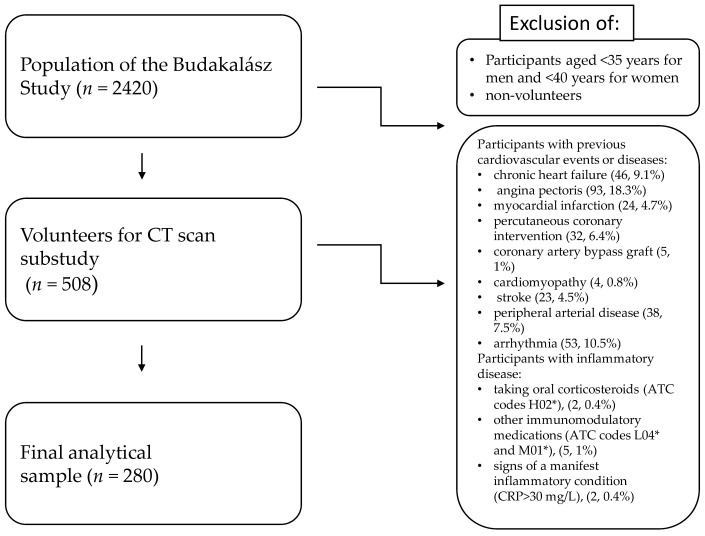
Study flowchart.

From the 2420 participants of the Budakalász Study, 508 persons volunteered for low-dose CT scan. Participants (*n*, %) with previous cardiovascular events or diseases such as chronic heart failure (46, 9.1%), angina pectoris (93, 18.3%), myocardial infarction (24, 4.7%), PCI (32, 6.4%), CABG (5, 1.0%), cardiomyopathy (4, 0.8%), stroke (23, 4.5%), PAD (38, 7.5%), or arrhythmia (53, 10.5%) and participants taking oral corticosteroids (ATC codes H02*, 2, 0.4%) or other immunomodulatory medications (ATC codes L04* and M01*, 5, 1%), and those with signs of a manifest inflammatory condition (CRP > 30 mg/L, 2, 0.4%), were excluded from further analysis, leaving 280 participants for the final analytical sample (Figure 1). As none of the participants came to their appointment with recent or acute infection, and none of them reported a haematological disease, these were not considered as specific exclusion criteria.

### 2.1. Baseline Characteristics by Dichotomized CACS

As expected, participants with higher CACS were older (mean age 57.4 (10.6) vs. 67 (7.6), *p* < 0.001), and more likely to be male (38.3% vs. 57%, *p* = 0.005). In addition, hypertension (42.8% vs. 81.0%, *p* < 0.001) and diabetes mellitus (5.5% vs. 27.8%, *p* < 0.001) were more frequent; and mean BMI (27.6 (4.8) vs. 29.2 (5.29), *p* = 0.02), NLR (1.88 (0.71) vs. 2.19 (0.88, *p* = 0.008) and HbA1c (5.75 (0.55) vs. 6.17 (1.1), *p* < 0.01) were significantly higher in those with a higher CACS (Table 1).

### 2.2. Baseline Characteristics by VAI Tertiles

The cut-off values for the sex-specific VAI tertiles were 1.367 and 1.438 for males and 1.942 and 2.048 for females.

The mean age was 60.3 (10.7) years. Proportion of males was 43.6%. Mean VAI values were 1.72 (0.3), 1.87 (0.3) and 1.9 (0.35) in the three groups, respectively. Frequency of hypertension and hyperlipidaemia, and the proportion of the sexes, did not differ significantly between the groups; mean BMI and high-sensitive C-reactive protein (hsCRP) levels were also similar in the VAI tertile groups (Table 2).

There was a significant difference between the groups regarding age: it gradually increased across the groups (means 56.8 (11.0), 61.6 (10.5) and 62.1 (10.1), respectively, *p* < 0.001).

The prevalence of diabetes mellitus was significantly higher in the 3^rd^ tertile group than in the other tertiles (21.5% vs. 7.4% and 6.5%, *p* = 0.002). HbA1c% level gradually increased across the groups (means 5.72 (0.58), 5.84 (0.65) and 6.04 (1.01), respectively, *p* = 0.02). Proportion of smokers was highest in the 1st tertile and lowest in the 2nd tertile (ratio of smokers: 18.3%, 7.4%, 10.8%, respectively, *p* = 0.067).

The ratio of those who had a coronary artery calcium score above 100 increased significantly through the VAI tertile groups (18.3%, 27.7% and 38.7%, respectively, *p* = 0.008).

### 2.3. Association of Neutrophil-to-Lymphocyte Ratio and Coronary Calcium Score across VAI Tertiles

While there was no significant difference in NLR values between the CACS groups in the lower 2 VAI tertiles, NLR was significantly lower in the low-risk CACS (≤100) compared to the moderate to high-risk CACS ( >100) group in the 3rd VAI tertile (*p* = 0.008) (Table 3).

### 2.4. Independent Determinants of Increased Coronary Artery Calcium Score

We performed multiple logistic regression analysis adjusted for age and sex (model 1); model 1 plus NLR and VAI interaction by VAI tertile (model 2); model 2 plus BMI and smoking (model 3); model 3 plus cardiometabolic diseases (hyperlipidaemia, hypertension, diabetes mellitus—model 4); and finally model 4 plus HbA1c and CRP (model 5) as covariates (Table 4).

Based on the nested multiple logistic regression models, NLR remained an independent determinant of the presence of coronary calcification (OR = 1.67, *p* = 0.03) in the 3rd VAI tertile, while no association in the other two tertiles was found. Older age, male sex, current smoking, and hypertension were also independently associated with elevated CACS. These results were further confirmed by the findings in the sensitivity analysis (data available on request).

## 3. Discussion

In the present study we examined the association between NLR and CACS in asymptomatic patients across VAI tertiles. We found a significant interaction between NLR and VAI tertiles: there was no association between NLR and increased CACS in the lower two VAI tertiles, but higher NLR was associated with an increased risk of elevated CACS in the highest VAI tertile. This association remained even when we adjusted for other important determinants of CACS such as age, sex, current smoking, and hypertension.

The literature seems to be equivocal in terms of an association of subclinical atherosclerosis, markers of adiposity, and NLR. Some studies have found that VAI and NLR are independent determinants of subclinical atherosclerosis in European [23] and Chinese populations [24]. Furthermore, the latter study also showed that when VAI and NLR are added to the ASCVD risk model, its goodness-of-fit and discriminability improved. However, another working group [25] reported on a significant association between NLR and the presence of non-calcified and mixed plaques but found no association with calcified plaques. Similarly, Nam et al. [26], in a population of 1000 healthy Korean adults, described an independent correlation between NLR and CACS only in men.

Serrano et al. [27] reported that NLR was independently associated with a CACS above 100 in asymptomatic patients. While we found no overall association between NLR and CACS, we think that the different findings could be explained by the different characteristics of the study populations. Our patient population did not include people with manifest cardiovascular disease (such as myocardial infarction or other vascular events in their medical history), and thus extends the role of NLR to a truly asymptomatic population.

Given that coronary artery calcium score is an important surrogate of later vascular events, and plays an important role in cardiovascular risk stratification, our results could be important for the improvement of cardiovascular risk estimation in asymptomatic persons. The pathomechanism of CACS can be linked to NLR at several points.

Age and male gender are non-modifiable risk factors that are known to be related to the extent of coronary calcification. A progressive increase in CACS with age is observed [28]. Smoking is a well-established risk factor for cardiovascular disease and because of its systemic effects it has a great impact on coronary plaque burden. In a former study, we have found that smoking is strongly associated with severe coronary calcification [29]. Previously, we have found that highest VAI tertile is an independent predictor for the presence of CACS > 100 in males [8]. There are some potential underlying mechanisms explaining the association between NLR and CACS in centrally obese patients.

Obesity and metabolic syndrome are both accompanied by a low-grade, chronic, systemic inflammation [30]. Increasing NLR level signals the presence of inflammation. On that basis, various studies showed a link between NLR, obesity, and metabolic disorders [31,32].

Central obesity is an important component of metabolic syndrome. Besides the presence of excess adipose tissue, its functioning is also altered. Secretion of pro-inflammatory adipokines is upregulated causing the activation of sympathetic nervous and renin angiotensin systems that further amplifies the inflammatory response. Moreover, impaired glucose metabolism is associated with insulin resistance. It can be characterized by increased fatty acid oxidation at the cellular level which leads to oxidative stress and indirectly to vascular inflammation. Over time, as these metabolic alterations are constantly present, the inflammation also becomes chronic [33].

Hypercholesterolemia is also known to lead to and sustain vascular inflammation [34]. Metabolic changes such as hyperglycaemia [35] and hypercholesterolaemia induce a pathway of atherosclerosis that involves neutrophils as well. Flynn et al. [36] have shown that fluctuations in blood glucose may cause an increase in circulating leukocytes, including those that promote atherosclerosis.

In line with these mechanisms, the prevalence of diabetes, and HbA1c and NLR levels, were higher in the 3rd VAI tertile in our study, indicating that hyperglycaemia is an important characteristic of this high-VAI group.

Different immune cells play roles in all stages of the atherosclerotic process [37]. Early leukocyte recruitment is linked to vascular endothelial dysfunction. Neutrophils are known to invade atherosclerotic plaques. Then, they may attract further immune cells, and promote oxidative stress and LDL modification. As neutrophils act toward atherosclerosis, lymphocytes have an opposite effect, they reduce the progression [38]. Therefore, if the neutrophil-to-lymphocyte ratio increases, it is a sign of progression. Furthermore, neutrophils take part in the destabilization of the plaques [39].

Calcification in an atherosclerotic plaque presents in two forms: microcalcification and macrocalcification [40]. Microcalcification is usually seen in earlier stages of atherosclerosis, furthermore, it activates the inflammatory response and is characteristic of an unstable plaque [41,42]. Over time, sheet-like, dense calcification develops that is observed mostly in stable, fibrocalcific plaques. There seems to be a paradox, because a high calcium score measured by CT is associated with a higher rate of cardiovascular events. Nevertheless, calcium score rather implies the total plaque burden and the highly calcified plaques are not the direct cause of the future events.

To the best of our knowledge, this is the first study demonstrating an association between NLR and coronary calcification specifically in centrally obese patients. The clinical significance of our findings on the enhanced role of subclinical inflammation among centrally obese people in subclinical atherosclerosis is highlighted by the high and increasing prevalence of central obesity in the general population. Furthermore, detailed phenotyping of our participants allowed us to investigate the independent association between VAI and subclinical atherosclerosis. NLR is an easily obtainable measure and its components may be readily available in electronic health records, suggesting its potential future clinical application in risk stratification or screening of asymptomatic individuals. However, this requires further exploration in longitudinal studies.

In conclusion, we have found that NLR is independently associated with coronary calcification in the group with the highest degree of visceral adiposity in an asymptomatic population. Our results suggest that VAI is not just a simple indicator of visceral adipose tissue dysfunction but could be a useful clinical marker of cardiometabolic risk and could have an effect on systemic inflammation. The association between VAI, NLR, and CACS may have significant implications for identifying patients at risk for atherosclerotic coronary artery disease in primary prevention.

## 4. Materials and Methods

The participants of the current study were recruited from a cardiovascular screening program (Budakalász Health Examination Survey) performed in a Central Hungarian town (Budakalász) between 2011 and 2013 [43]. Volunteers (≥35 years of age for men and ≥40 years for women) underwent a low-dose (approx. 0.5 mSv) cardiac CT scan (Brilliance iCT, Philips Healthcare, Best, The Netherlands). If necessary, participants received oral beta-blockers. Offline semi-automatic software analysis (Calcium scoring, Heartbeat-CS, Philips Healthcare) was used to calculate the Agatston score, calcification area, and volume. For the current analysis, Agatston scores were dichotomized (≤100 vs. > 100) [44].

Information on medical history, anthropometric data, and blood pressure were collected using standardized procedures.

Among the lifestyle measures, we defined smoking as currently smoking at least one cigarette per day.

Blood pressure was measured with an automated machine in triplicate. The mean of the second and third was used as measured blood pressure. We defined hypertension as self-report of doctor-diagnosed hypertension, regular use of blood-pressure-lowering medication, or measured blood pressure ≥ 140/90 mmHg. Diabetes mellitus was defined as self-report of doctor-diagnosed diabetes, regular use of antidiabetic medication, or an HbA1c ≥ 6.5%. Hyperlipidaemia was defined as self-report of doctor-diagnosed hyperlipidaemia or regular use of lipid-lowering medication (e.g., statin, ezetimibe, fibrate).

In the current analysis we used body height (measured to the nearest 0.1 cm on a stadiometer), weight (measured in light clothing), and waist circumference (measured halfway between the ribcage and the iliac crest). Body mass index was calculated by the standard Quetelet’s formula.

Laboratory tests were performed in the Central Laboratory of Semmelweis University. For the current analysis, we used differential blood cell counts, HbA1c, serum creatinine, lipid profile (high-density lipoprotein [HDL], triglyceride [TG]), and high-sensitivity C-reactive protein (hsCRP) levels.

VAI was calculated according to Amato et al. [4] using the following sex-specific formula:

- Males: VAI = [WC/{39.68 + (1.88 × BMI)}] × (TG/1.03) × (1.31/HDL-C)
- Females: VAI = [WC/{36.58 + (1.89 × BMI)}] × (TG/0.81) × (1.52/HDL-C)

Then the VAI values were ranked separately for men and women, and sex-specific tertiles were created for further analysis.

NLR was calculated as the ratio of the absolute neutrophil count to the absolute lymphocyte count from the differential blood cell count [45].

### Statistical Methods

Descriptive statistics are given as means ± standard deviation (SD) for continuous and *n* (%) for categorical variables. Normality of continuous variables was checked by visual inspection of histograms and normality plots and formally by normality tests. Variables with skewed distribution were log-transformed to improve normality.

Differences in baseline characteristics by low and high CACS score were tested by independent sample t-tests for continuous and by chi-squared tests for categorical variables.

Differences in baseline characteristics between VAI tertiles were tested by one-way ANOVA for continuous and by chi-squared tests for categorical variables. P values are given for both heterogeneity and for linear contrasts over VAI tertiles.

Next, we ran a set of nested, hierarchical multiple logistic regression models with dichotomized CACS as the outcome adjusted for age and sex (Model 1); Model 1 + NLR by VAI tertile interaction (Model 2); Model 2 + BMI and smoking (Model 3); Model 3 + cardiometabolic diseases (hyperlipidaemia, hypertension, diabetes mellitus—Model 4); and finally Model 4 + HbA1c and CRP (Model 5) as covariates (Table 4). This setting allowed the investigation of the role of NLR in each VAI tertile using all available data (i.e., all 280 participants).

Finally, we repeated the multiple logistic regression analysis without exclusion of participants taking oral corticosteroids (ATC codes H02*) or other immunomodulatory medications (ATC codes L04* and M01*) and those with signs of a manifest inflammatory condition (CRP ≤ 30 mg/L as a sensitivity analysis.

IBM SPSS Statistics (version 27.0, IBM, Armonk, NY, USA) was used for all statistical analyses. Statistical significance was set at two tailed *p* values < 0.05.

## 5. Limitations

This study has some limitations. First, as in any cross-sectional study NLR and CACS were measured at the same time; that limits our ability to investigate a cause–effect relationship. Second, we offered low-dose CT only for men above 35 and women above 40, therefore patients with the lowest cardiovascular risk were excluded from our study. Moreover, patients with former cardiovascular events were also excluded as Ca-score screening is suggested only in primary prevention. Third, we did not have information on history of chronic inflammatory disease or anti-inflammatory drug consumption.

## Figures and Tables

**Table 1:** Demographic data and risk factors in the study population divided into groups with coronary artery calcium score above and below 100.

Demographics and Risk Factors (*n* (%)/Mean (SD))	CACS ≤ 100 (201)	CACS > 100 (79)	*p*
BMI, mean (SD)	27.6 (4.8)	29.2 (5.29)	0.02
hsCRP, mean (SD)	2.63 (3.05)	2.89 (3.68)	NS
HbA1c, mean (SD)	5.75 (0.55)	6.17 (1.1)	0.01
age, mean (SD)	57.4 (10.6)	67 (7.6)	<0.001
NLR, mean (SD)	1.88 (0.71)	2.19 (0.88)	0.008
smoking, *n* (%)	24 (11.9)	10 (12.7)	NS
HT, *n* (%)	86 (42.8)	64 (81.0)	<0.001
HLP, *n* %	74 (36.8)	38 (48.1)	NS
DM, *n* %	11 (5.5)	22 (27.8)	<0.001
Sex, *n* %	77 (38.3)	45 (57.0)	0.005

BMI—body mass index, CACS—coronary artery calcium score, DM—diabetes mellitus, HbA1c—haemoglobin A1C, hsCRP—high-sensitivity C-reactive protein, HLP—hyperlipidaemia, HT—hypertension, NLR—neutrophil-to-lymphocyte ratio, NS—nonsignificant, SD—standard deviation (*p* stands for chi square test and *t*-test according to the type of variables).

**Table 2:** Demographic data and vascular risk factors in the study population overall and by VAI tertiles.

Demographics and Risk Factors (*n* (%)/Mean (SD)	Total Population	VAI 1st Tertile	VAI 2nd Tertile	VAI 3rd Tertile	*p*
*n* (%)	280 (100)	93	94	93	NS
Male, *n* (%)	122 (43.6)	41 (42.7)	42 (43.3)	41 (42.7)	NS
VAI mean (SD)	1.73 (0.32)	1.72 (0.3)	1.87 (0.3)	1.9 (0.35)	<0.001
HLP, *n* (%)	112 (40.8)	33 (35.5)	39 (41.5)	40 (43.0)	NS
HT, *n* (%)	150 (53.6)	41 (44.1)	54 (57.4)	55 (59.1)	NS
DM, *n* (%)	33 (11.8)	6 (6.5)	7 (7.4)	20 (21.5)	0.002
Smoking, *n* (%)	34 (12.1)	17 (18.3)	7 (7.4)	10 (10.8)	NS
Age, years, mean (SD)	60.3 (10.7)	56.8 (11.0)	61.6 (10.5)	62.1 (10.1)	0.001
BMI, (kg/m^2^), mean (SD)	28.1 (5.0)	27.5 (5.3)	29.0 (4.3)	27.7 (5.2)	NS
NLR, mean (SD)	1.97 (0.77)	1.88 (0.66)	1.9 (0.71)	2.14 (0.91)	0.042
HbA1c, %, mean (SD)	5.87 (0.78)	5.72 (0.58)	5.84 (0.65)	6.04 (1.01)	0.02
hsCRP, mmol/L, mean (SD)	2.71 (3.24)	2.43 (3.26)	2.65 (2.8)	3.05 (3.6)	NS
CACS > 100, *n* (%)	79 (28.2)	17 (18.3)	26 (27.7)	36 (38.7)	0.008
CACS, mean (SD)	174 (448)	98.5 (221)	188.1 (469)	236 (572)	NS

BMI—body mass index, CACS—coronary artery calcium score, DM—diabetes mellitus, HbA1c—haemoglobin A1C, hsCRP—high-sensitivity C-reactive protein, HLP—hyperlipidaemia, HT—hypertension, NLR—neutrophil-to-lymphocyte ratio, NS—nonsignificant, SD—standard deviation, VAI—visceral adiposity index, (*p* stands for chi square test and ANOVA test according to the type of variables).

**Table 3:** Comparing neutrophil-to-lymphocyte ratio in patients with coronary artery calcium score above and below 100 in the VAI tertile groups.

NLR-Mean	Low Risk (CACS ≤ 100)	Moderate to High Risk (CACS > 100)	*p*
VAI 1st tertile	1.86 (0.67)	1.94 (0.59)	NS
VAI 2nd tertile	1.89 (0.73)	1.94 (0.66)	NS
VAI 3rd tertile	1.89 (0.74)	2.48 (1.1)	0.008

CACS—coronary artery calcium score, NLR—neutrophil-to-lymphocyte ratio, NS—nonsignificant, VAI—visceral adiposity index, *p* values stand for *t*-tests.

**Table 4:** Predictors of increased CACS—nested models.

	Model 1			Model 2			Model 3			Model 4			Model 5		
	OR	95% CI	*p*	OR	95% CI	*p*	OR	95% CI	*p*	OR	95% CI	*p*	OR	95% CI	*p*
Male	2.70	1.48–4.93	0.00	2.61	1.4–4.85	0.00	2.42	1.29–4.53	0.01	2.08	1.06–4.07	0.03	2.05	1.03–4.08	0.04
Age	1.13	1.08–5.03	<0.001	1.12	1.08–1.16	<0.001	1.13	1.08–1.17	<0.001	1.11	1.06–1.17	<0.001	1.11	1.06–1.16	<0.001
VAI tertile 1 by NLR				0.99	0.60–1.59	NS	1.06	0.65–1.72	0.03	1.09	0.67–1.72	NS	1.16	0.7–1.92	NS
VAI tertile 2 by NLR				1.09	0.687–1.75	NS	1.13	0.71–1.82	NS	1.13	0.69–1.86	NS	1.21	0.73–1.99	NS
VAI tertile 3 by NLR				1.45	0.97–2.18	0.07	1.56	1.02–2.4	0.04	1.52	1–2.34	0.05	1.67	1.06–2.62	0.03
Current smoking							2.18	0.82–5.7	NS	3.20	1.2–10.45	0.02	3.97	1.34–11.7	0.02
BMI (kg/m^2^)							1.08	1–1.15	0.02	1.04	0.97–1.1	NS	1.05	0.97–1.14	NS
HLP										1.62	0.83–3.15	NS	1.50	0.79–3.3	NS
HT										3.14	1.4–6.87	0.01	3.14	1.43–6.91	<0.01
DM										2.25	0.85–5.95	NS	1.69	1.73–4.93	NS
HbA1c (%)													1.28	0.81–2.04	NS
hsCRP (mg/L)													0.93	0.82–1.05	NS

BMI—body mass index, CACS—coronary artery calcium score, DM—diabetes mellitus, HbA1c—haemoglobin A1C, hsCRP—high-sensitivity C-reactive protein, HLP—hyperlipidaemia, HT—hypertension, NLR—neutrophil-to-lymphocyte ratio, NS—nonsignificant, VAI—visceral adiposity index.

## Data Availability

The data that support the findings of this study are available from the corresponding author upon reasonable request.

## References

[1] Townsend N., Nichols M., Scarborough P., Rayner M. (2015). Cardiovascular disease in Europe—Epidemiological update 2015. Eur. Heart J..

[2] Mitchell J.D., Paisley R., Moon P., Novak E., Villines T.C. (2018). Coronary Artery Calcium and Long-Term Risk of Death, Myocardial Infarction, and Stroke: The Walter Reed Cohort Study. JACC Cardiovasc. Imaging.

[3] Rijlaarsdam-Hermsen D., Lo-Kioeng-Shioe M.S., Kuijpers D., van Domburg R.T., Deckers J.W., van Dijkman P.R.M. (2020). Prognostic value of the coronary artery calcium score in suspected coronary artery disease: Amstudy of 644 symptomatic patients. Neth. Heart J. Mon. J. Neth. Soc. Cardiol. Neth. Heart Found..

[4] Amato M.C., Giordano C., Galia M., Criscimanna A., Vitabile S., Midiri M., Galluzzo A. (2010). Visceral Adiposity Index: A reliable indicator of visceral fat function associated with cardiometabolic risk. Diabetes Care.

[5] Iliodromiti S., Celis-Morales C.A., Lyall D.M., Anderson J., Gray S.R., Mackay D.F., Nelson S.M., Welsh P., Pell J.P., Gill J.M.R. (2018). The impact of confounding on the associations of different adiposity measures with the incidence of cardiovascular disease: A cohort study of 296 535 adults of white European descent. Eur. Heart J..

[6] Qiao T., Luo T., Pei H., Yimingniyazi B., Aili D., Aimudula A., Zhao H., Zhang H., Dai J., Wang D. (2022). Association between abdominal obesity indices and risk of cardiovascular events in Chinese populations with type 2 diabetes: A prospective cohort study. Cardiovasc. Diabetol..

[7] Kouli G.M., Panagiotakos D.B., Kyrou I., Georgousopoulou E.N., Chrysohoou C., Tsigos C., Tousoulis D., Pitsavos C. (2017). Visceral adiposity index and 10-year cardiovascular disease incidence: The ATTICA study. Nutr. Metab. Cardiovasc. Dis. NMCD.

[8] Bagyura Z., Kiss L., Lux Á., Csobay-Novák C., Jermendy Á.L., Polgár L., Szelid Z., Soós P., Merkely B. (2020). Association between coronary atherosclerosis and visceral adiposity index. Nutr. Metab. Cardiovasc. Dis. NMCD.

[9] Maréchal P., Tridetti J., Nguyen M.L., Wéra O., Jiang Z., Gustin M., Donneau A.F., Oury C., Lancellotti P. (2020). Neutrophil Phenotypes in Coronary Artery Disease. J. Clin. Med..

[10] Cooper H.A., Exner D.V., Waclawiw M.A., Domanski M.J. (1999). White blood cell count and mortality in patients with ischemic and nonischemic left ventricular systolic dysfunction (an analysis of the Studies Of Left Ventricular Dysfunction [SOLVD]). Am. J. Cardiol..

[11] Warny M., Helby J., Nordestgaard B.G., Birgens H., Bojesen S.E. (2020). Incidental lymphopenia and mortality: A prospective cohort study. CMAJ Can. Med. Assoc. J. = J. De L’association Med. Can..

[12] Rodríguez-Rodríguez E., López-Sobaler A.M., Ortega R.M., Delgado-Losada M.L., López-Parra A.M., Aparicio A. (2020). Association between Neutrophil-to-Lymphocyte Ratio with Abdominal Obesity and Healthy Eating Index in a Representative Older Spanish Population. Nutrients.

[13] Karakaya S., Altay M., Kaplan Efe F., Karadağ İ., Ünsal O., Bulur O., Eser M., Taner Ertuğrul D. (2019). The neutrophil-lymphocyte ratio and its relationship with insulin resistance in obesity. Turk. J. Med. Sci..

[14] Angkananard T., Anothaisintawee T., McEvoy M., Attia J., Thakkinstian A. (2018). Neutrophil Lymphocyte Ratio and Cardiovascular Disease Risk: A Systematic Review and Meta-Analysis. BioMed Res. Int..

[15] Li W., Hou M., Ding Z., Liu X., Shao Y., Li X. (2021). Prognostic Value of Neutrophil-to-Lymphocyte Ratio in Stroke: A Systematic Review and Meta-Analysis. Front. Neurol..

[16] Wang Z., Wang J., Cao D., Han L. (2020). Correlation of neutrophil-to-lymphocyte ratio with the prognosis of non-ST-segment elevation in patients with acute coronary syndrome undergoing selective percutaneous coronary intervention. J. Int. Med. Res..

[17] Dentali F., Nigro O., Squizzato A., Gianni M., Zuretti F., Grandi A.M., Guasti L. (2018). Impact of neutrophils to lymphocytes ratio on major clinical outcomes in patients with acute coronary syndromes: A systematic review and meta-analysis of the literature. Int. J. Cardiol..

[18] Del Turco S., Bastiani L., Minichilli F., Landi P., Basta G., Pingitore A., Vassalle C. (2022). Interaction of Uric Acid and Neutrophil-to-Lymphocyte Ratio for Cardiometabolic Risk Stratification and Prognosis in Coronary Artery Disease Patients. Antioxidants.

[19] Dziedzic E.A., Gąsior J.S., Tuzimek A., Dąbrowski M., Jankowski P. (2022). Neutrophil-to-Lymphocyte Ratio Is Not Associated with Severity of Coronary Artery Disease and Is Not Correlated with Vitamin D Level in Patients with a History of an Acute Coronary Syndrome. Biology.

[20] Taurino M., Aloisi F., Del Porto F., Nespola M., Dezi T., Pranteda C., Rizzo L., Sirignano P. (2021). Neutrophil-to-Lymphocyte Ratio Could Predict Outcome in Patients Presenting with Acute Limb Ischemia. J. Clin. Med..

[21] Spark J.I., Sarveswaran J., Blest N., Charalabidis P., Asthana S. (2010). An elevated neutrophil-lymphocyte ratio independently predicts mortality in chronic critical limb ischemia. J. Vasc. Surg..

[22] Pasqui E., de Donato G., Lazzeri E., Molino C., Galzerano G., Giubbolini M., Palasciano G. (2022). High Neutrophil-to-Lymphocyte and Platelet-to-Lymphocyte Ratios Are Associated with a Higher Risk of Hemodialysis Vascular Access Failure. Biomedicines.

[23] Suárez-Cuenca J.A., Ruíz-Hernández A.S., Mendoza-Castañeda A.A., Domínguez-Pérez G.A., Hernández-Patricio A., Vera-Gómez E., De la Peña-Sosa G., Banderas-Lares D.Z., Montoya-Ramírez J., Blas-Azotla R. (2019). Neutrophil-to-lymphocyte ratio and its relation with pro-inflammatory mediators, visceral adiposity and carotid intima-media thickness in population with obesity. Eur. J. Clin. Investig..

[24] Li B., Lai X., Yan C., Jia X., Li Y. (2020). The associations between neutrophil-to-lymphocyte ratio and the Chinese Visceral Adiposity Index, and carotid atherosclerosis and atherosclerotic cardiovascular disease risk. Exp. Gerontol..

[25] Li T., Gu C., Wang F., Lv B., Zhang C., Peng R., Cong X., Chen X. (2018). Association of Neutrophil-Lymphocyte Ratio and the Presence of Noncalcified or Mixed Coronary Atherosclerotic Plaques. Angiology.

[26] Nam S.H., Kang S.G., Song S.W. (2017). The Neutrophil-Lymphocyte Ratio Is Associated with Coronary Artery Calcification in Asymptomatic Korean Males: A Cross-Sectional Study. BioMed Res. Int..

[27] Serrano C.V., de Mattos F.R., Pitta F.G., Nomura C.H., de Lemos J., Ramires J.A.F., Kalil-Filho R. (2019). Association between Neutrophil-Lymphocyte and Platelet-Lymphocyte Ratios and Coronary Artery Calcification Score among Asymptomatic Patients: Data from a Cross-Sectional Study. Mediat. Inflamm..

[28] Nicoll R., Wiklund U., Zhao Y., Diederichsen A., Mickley H., Ovrehus K., Zamorano J., Gueret P., Schmermund A., Maffei E. (2016). Gender and age effects on risk factor-based prediction of coronary artery calcium in symptomatic patients: A Euro-CCAD study. Atherosclerosis.

[29] Kiss L.Z., Bagyura Z., Csobay-Novák C., Lux Á., Polgár L., Jermendy Á., Soós P., Szelid Z., Maurovich-Horvat P., Becker D. (2019). Serum Uric Acid Is Independently Associated with Coronary Calcification in an Asymptomatic Population. J. Cardiovasc. Transl. Res..

[30] Rohm T.V., Meier D.T., Olefsky J.M., Donath M.Y. (2022). Inflammation in obesity, diabetes, and related disorders. Immunity.

[31] Zhu Y., Li G., Laukkanen J.A., Song X., Zhang J., Wei L., Chen X., Li Y., Liu C. (2022). Higher neutrophil to lymphocyte ratio is associated with renal dysfunction and cardiac adverse remodeling in elderly with metabolic syndrome. Front. Cardiovasc. Med..

[32] Hashemi Moghanjoughi P., Neshat S., Rezaei A., Heshmat-Ghahdarijani K. (2022). Is the Neutrophil-to-Lymphocyte Ratio an Exceptional Indicator for Metabolic Syndrome Disease and Outcomes?. Endocr. Pract..

[33] Furukawa S., Fujita T., Shimabukuro M., Iwaki M., Yamada Y., Nakajima Y., Nakayama O., Makishima M., Matsuda M., Shimomura I. (2004). Increased oxidative stress in obesity and its impact on metabolic syndrome. J. Clin. Investig..

[34] Osaka M., Deushi M., Aoyama J., Funakoshi T., Ishigami A., Yoshida M. (2021). High-Fat Diet Enhances Neutrophil Adhesion in LDLR-Null Mice Via Hypercitrullination of Histone H3. JACC Basic Transl. Sci..

[35] Shah M.S., Brownlee M. (2016). Molecular and Cellular Mechanisms of Cardiovascular Disorders in Diabetes. Circ. Res..

[36] Flynn M.C., Kraakman M.J., Tikellis C., Lee M.K.S., Hanssen N.M.J., Kammoun H.L., Pickering R.J., Dragoljevic D., Al-Sharea A., Barrett T.J. (2020). Transient Intermittent Hyperglycemia Accelerates Atherosclerosis by Promoting Myelopoiesis. Circ. Res..

[37] Vallejo J., Cochain C., Zernecke A., Ley K. (2021). Heterogeneity of immune cells in human atherosclerosis revealed by scRNA-Seq. Cardiovasc. Res..

[38] Mauersberger C., Hinterdobler J., Schunkert H., Kessler T., Sager H.B. (2021). Where the Action Is-Leukocyte Recruitment in Atherosclerosis. Front. Cardiovasc. Med..

[39] Silvestre-Roig C., Braster Q., Ortega-Gomez A., Soehnlein O. (2020). Neutrophils as regulators of cardiovascular inflammation. Nat. Rev. Cardiol..

[40] Akers E.J., Nicholls S.J., Bartolo B.A.D. (2019). Plaque Calcification. Arterioscler. Thromb. Vasc. Biol..

[41] Jinnouchi H., Sato Y., Sakamoto A., Cornelissen A., Mori M., Kawakami R., Gadhoke N.V., Kolodgie F.D., Virmani R., Finn A.V. (2020). Calcium deposition within coronary atherosclerotic lesion: Implications for plaque stability. Atherosclerosis.

[42] Balmos I.A., Horváth E., Brinzaniuc K., Muresan A.V., Olah P., Molnár G.B., Nagy E.E. (2023). Inflammation, Microcalcification, and Increased Expression of Osteopontin Are Histological Hallmarks of Plaque Vulnerability in Patients with Advanced Carotid Artery Stenosis. Biomedicines.

[43] Bagyura Z., Kiss L., Edes E., Lux A., Polgár L., Soós P., Szenczi O., Szelid Z., Vadas R., Józan P. (2014). Cardiovascular screening programme in the Central Hungarian region. The Budakalász Study. Orv. Hetil..

[44] Grundy S.M., Stone N.J., Bailey A.L., Beam C., Birtcher K.K., Blumenthal R.S., Braun L.T., de Ferranti S., Faiella-Tommasino J., Forman D.E. (2019). 2018 AHA/ACC/AACVPR/AAPA/ABC/ACPM/ADA/AGS/APhA/ASPC/NLA/PCNA Guideline on the Management of Blood Cholesterol: A Report of the American College of Cardiology/American Heart Association Task Force on Clinical Practice Guidelines. Circulation.

[45] Buonacera A., Stancanelli B., Colaci M., Malatino L. (2022). Neutrophil to Lymphocyte Ratio: An Emerging Marker of the Relationships between the Immune System and Diseases. Int. J. Mol. Sci..

